# Establishing reference curves for vital tissue perfusion using quantitative near-infrared fluorescence imaging with indocyanine green

**DOI:** 10.1007/s00423-024-03589-1

**Published:** 2025-01-08

**Authors:** Floris P. Tange, Roderick C. Peul, Pim van den Hoven, Stefan Koning, Mo W. Kruiswijk, Robin A. Faber, Pieter S. Verduijn, Carla S. P. van Rijswijk, Hidde A. Galema, Denise E. Hilling, Sam P. J. van Dijk, Tessa M. van Ginhoven, Stijn Keereweer, Marc A. M. Mureau, Eline A. Feitsma, Milou E. Noltes, Schelto Kruijff, Caroline Driessen, Michael P. Achiam, Abbey Schepers, Jan van Schaik, J. Sven D. Mieog, Alexander L. Vahrmeijer, Jaap F. Hamming, Joost R. van der Vorst

**Affiliations:** 1 Department of Surgery, Leiden University Medical Center, Albinusdreef 2, 2333 ZA Leiden, The Netherlands; 2 Department of Plastic and Reconstructive Surgery, Leiden University Medical Center, Albinusdreef 2, 2333 ZA Leiden, the Netherlands; 3 Department of Interventional Radiology, Leiden University Medical Centre, Leiden, The Netherlands; 4 Department of Surgical Oncology and Gastrointestinal Surgery, Erasmus MC Cancer Institute, Doctor Molewaterplein 40, 3015 GD Rotterdam, the Netherlands; 5 Department of Otorhinolaryngology, Head and Neck Surgery, Erasmus MC Cancer Institute, Rotterdam, The Netherlands; 6 Department of Plastic and Reconstructive Surgery, Erasmus MC Cancer Institute, University Medical Center Rotterdam, Rotterdam, The Netherlands; 7 Department of Surgery, University Medical Center Groningen, Hanzeplein 1, 9713 GZ Groningen, The Netherlands; 8 Department of Plastic and Reconstructive Surgery, Amsterdam University Medical Center, Meibergdreef 9, 1105 AZ Amsterdam, the Netherlands; 9 Department of Surgery and Transplantation, Copenhagen University Hospital Rigshospitalet, The Capital Region of Denmark, Copenhagen, Denmark

**Keywords:** Near-infrared fluorescence, Indocyanine green, Quantification, Surgery, Tissue perfusion

## Abstract

**Purpose:**

Assessment of tissue perfusion using near-infrared fluorescence (NIR) with indocyanine green (ICG) is gaining popularity, however reliable and objective interpretation remains a challenge. Therefore, this study aimed to establish reference curves for vital tissue perfusion across target tissues using this imaging modality.

**Methods:**

Data from five prospective study cohorts conducted in three Dutch academic medical centres between December 2018 and June 2023 was included. Quantitative analysis using time-intensity curves was performed in ten target tissues, including the colon, ileum, gastric conduit, deep inferior epigastric artery perforator (DIEP) flap, skin of the foot, trachea, sternocleidomastoid muscle (SCM), carotid artery, parathyroid gland, and skin of the neck.

**Results:**

A total of 178 patients were included in this study, representing 303 target tissues. Three different patterns of reference curves were identified based on a subjective assessment. Seven out of ten tissues showed a reference curve with rapid inflow (median time-to-max (tmax): 13.0–17.8 s, median maximum-normalized-slope (slope norm): 10.6–12.6%/sec), short outflow (median area-under-the-curve of tmax + 60 s (AUC60): 65.0–85.1%) followed by a gradual/absent outflow. Secondly, the DIEP flap and SCM tissue showed a reference curve with longer inflow (median tmax: 24.0, 22.0 s, median slope norm: 9.3, 9.7%/sec respectively) and reduced outflow (median AUC60: 89.1, 89.0% respectively). Thirdly, the skin of the foot showed slow inflow (median tmax 141.1 s, median norm slope 2.1%/sec) without outflow.

**Conclusion:**

This study demonstrates reference curves for vital tissue perfusion of multiple target tissues identified with ICG NIR fluorescence imaging, providing a critical step towards the clinical implementation of this technique.

## Introduction

Adequate assessment of tissue perfusion is a critical part of surgery and heavily influences patient outcomes [1–4]. Comprised tissue perfusion can lead to severe complications such as impaired wound healing, necrosis, and organ dysfunction [5–11]. Traditionally, tissue perfusion assessment has predominantly relied on clinical judgement including visual inspection, palpation, and tissue temperature. While clinical judgement remains indispensable in surgical decision-making, it is a subjective method that often lacks predictive accuracy [12–14]. Moreover, surgical procedures are increasingly performed minimally invasive, impeding adequate assessment [15]. To reduce the risk of undesired outcomes related to malperfusion, the implementation of a more objective modality for perioperative perfusion assessment can offer significant clinical value [5, 16, 17]. One of these modalities is near-infrared (NIR) fluorescence imaging with indocyanine green (ICG), a valuable technique that is already being used extensively in various surgical fields [9, 17–25]. This technique uses the contrast dye ICG which can be visualised by a NIR camera system upon intravenous injection, allowing real-time perfusion assessment. This imaging modality has been widely evaluated in the literature, including large, randomized trials, often by using a qualitative and therefore subjective interpretation of the fluorescence signal [26–28]. In recent years, studies focusing on quantification of the fluorescence intensity are emerging, supported by the increasing availability of dedicated software programs for this purpose. For various target tissues, these studies have shown that quantification of the fluorescence significantly improves the validity and reliability of ICG NIR fluorescence imaging [22, 24, 29–31]. However, the high variability of utilized quantification methods impedes the clinical usefulness and hampers adequate interpretation of actual perfusion [24, 32]. The results however always focus on the interpretation of time-intensity curves. Moreover, it is essential to establish reference curves of perfusion for vital tissue.

These reference curves could serve as a threshold for the differentiation between adequately and inadequately perfused tissue [20, 22, 24, 29, 30, 33]. As an initial step, this study aims to explore the reference curves of adequate perfusion in multiple vital target tissues.

## Material and methods

Data originating from five separately designed prospective study cohorts conducted in three Dutch academic centres was pooled. The included tissue types and imaging protocols of each study cohort are described in Table 1. Each study cohort was approved by the respective medical ethics committee and was performed according to the declaration of Helsinki (10th version, Fortaleza, 2013). All patients provided informed consent. Each study cohort used ICG VERDYE (25 mg vial, Diagnostic Green GmbH, Aschheim-Dornach, Germany) which was intravenously injected, directly followed by a saline flush. The fluorescence videos were all recorded with the Quest Spectrum 2.0 camera system (Quest Medical Imaging, Middenmeer, the Netherlands).

**Table 1:** Imaging protocols of study cohorts

**Included tissues**	**Study cohorts**	Camera system	Camera distance (cm)	Camera settings (gain/exposure)	ICG dosage	Duration fluorescence recordings (min)
o Colon o Ileum	Colorectal resection [38]	Quest Spectrum 2.0	30	20.0 dB/ 50 ms	5 mg	5
Gastric conduit	Oesophagectomy – gastric conduit reconstruction [39]	Quest Spectrum 2.0	30	20.0 dB/ 100 ms	5 mg	5
DIEP flap	DIEP flap [40]	Quest Spectrum 2.0	60	21.6 dB/ 200 ms	7.5 mg	2 or 4
Skin foot	Skin foot [15]	Quest Spectrum 2.0	50	20.0 dB/ 145 ms	0.1 mg/kg	10
o Trachea o SCM o Carotid artery o Parathyroid glands o Skin neck	Total thyroidectomy	Quest Spectrum 2.0	30	22.5 dB/ 50 ms	1.5 mg/ 1L blood volume	2.5

Abbreviations: ICG, indocyanine green, DIEP, deep inferior epigastric perforator, SCM, sternocleidomastoid muscle

### Data collection

The data for this study was collected from December 2018 to June 2023. For the colorectal resection and the gastric conduit cohort, the data was acquired according to previously described study protocols [29, 30]. The data from the DIEP flap cohort originates from an ongoing randomised controlled trial (NCT05507710) and was acquired according to the published trial protocol [34]. Data from the skin foot study cohort originates from the control group of a previously published study by van den Hoven et al*.* [14]. The total thyroidectomy cohort is an ongoing study from which the included data was collected (UMCG research register: 201,900,307). The fluorescence imaging was performed directly after the total thyroid gland was resected.

### Data analysis

**Fig. 1:**
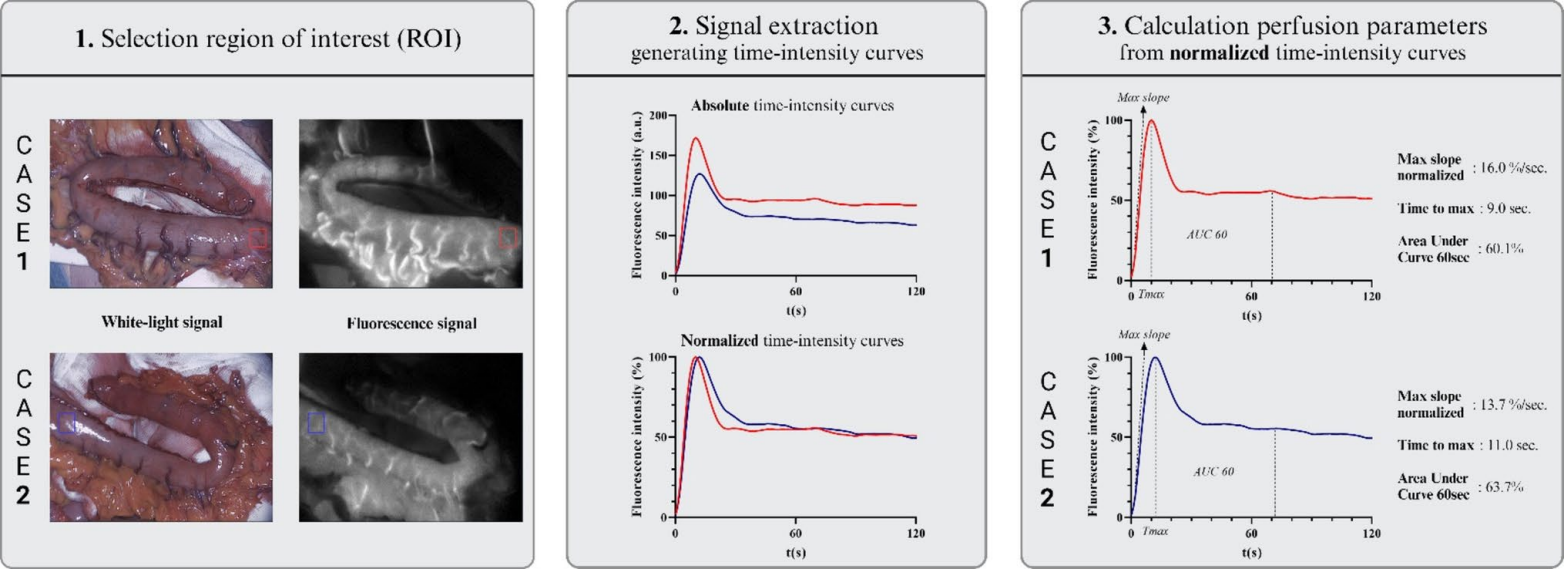
Fluorescence signal quantification explained: establishment of time-intensity curves and perfusion parameters in two example cases. Abbreviations: t, time, a.u., arbitrary units

All acquired fluorescence videos were analysed using the Quest Research Framework® software (Quest Medical Imaging, Middenmeer, the Netherlands). An overview of the signal quantification workflow in two example cases is visualized in Fig. 1. The ROIs for this study were systematically defined by the first author for each tissue type to ensure consistency, independent of previously used ROI’s of each study cohort. For each tissue, the ROI placement was guided by clinical assessment (expert surgeon opinion) of perfusion adequacy and aimed to capture the most representative regions. If there was uncertainty in the clinical assessment of vitality, tissue ROIs were excluded. In the colon and ileum groups, the ROI was located most distally from the resection plane, or on a visual part of the colon/ileum which was not operated on. The selected ROI of the gastric conduit was located at the most proximal part of the gastric conduit (nearest to the pylorus). The selected DIEP flap ROI directly surrounded the location where the perforator artery entered the flap. The ROI of the skin foot group consisted of the total dorsum of the foot, including the toes. The selected ROI for the skin of the neck group was located dorsal from the incision wound. The selected ROI on the sternocleidomastoid muscle (SCM) was located at the best visual part of the muscle. For the carotid artery, the ROI comprised the total visual artery. The ROI of the trachea was set at the complete visual trachea. In the parathyroid gland group, the included patients did not suffer from postoperative hypoparathyroidism. The parathyroid glands were identified by the attending surgeon and all visual glands were selected as ROIs.

The fluorescence signal from each ROI was quantified into a time-intensity curve, displaying the fluorescence intensity over time. Both absolute and normalized time-intensity curves were generated, and three parameters were calculated from the normalized time-intensity curves, including the time to maximum intensity (Tmax), maximum normalized slope (slope norm) and the area under the curve + 60 s after Tmax (AUC60).

Normalization of the time-intensity curves was performed according to a previously described method [35]. Baseline subtraction, motion tracking and a moving average filter were applied in the quantification software. All fluorescence videos were analysed for two minutes. To analyse the perfusion patterns, the curves were subjectively categorized into three distinct patterns based on visual assessment, parameter values and expert judgement.

The Kruskal–Wallis test was employed for an overall comparison of Tmax, Slope norm, and AUC60 values across the three identified perfusion pattern groups. To examine differences between each pair of patterns within the three perfusion parameters, pairwise comparisons were conducted using the Mann–Whitney U-test. A p-value of less than 0.05 was considered statistically significant for all tests. Statistical analysis was performed using IBM SPSS Statistics 29.0 (IBM SPSS Statistics for Windows, Version 29.0. Armonk, NY: IBM Corp).

## Results

### Patient characteristics

A total of 178 patients (303 ROIs) were included in this study. Baseline patient characteristics are presented in Table 2. The mean age in the tissue groups ranged from 38.3 to 66.6 years and the mean body mass index ranged from 25.3 to 28.4. The proportion of females in the DIEP flap group was 100% and ranged from 13.2 to 93.3% in the other nine tissue groups. The colorectal resection cohort consisted of 30 patients, from which colon perfusion was analysed in 29 patients (30 ROIs) and ileum perfusion in 11 patients (11 ROIs). The gastric conduit cohort consisted of 53 patients. Nineteen patients undergoing a DIEP flap reconstruction were included, in which 29 perforators were identified. The skin foot cohort consisted of 16 patients with a total of 32 included feet. The total thyroidectomy cohort consists of 60 patients. From these patients, 52 ROIs of skin in the neck region, 44 ROIs of the trachea, 13 ROIs of the sternocleidomastoid muscle and 15 ROIs of the carotid artery were included. The parathyroid gland group consisted of 10 patients with 24 identified parathyroid glands.

**Table 2:** Patient characteristics

	**Colon** (n = 29)	**Ileum** (n = 11)	**Gastric conduit** (n = 53)	**DIEP flap** (n = 19)	**Skin foot** (n = 16)	**Trachea** (n = 44)	**SCM** (n = 13)	**Carotid artery** (n = 15)	**Parathyroid gland** (n = 10)	**Skin neck** (n = 52)
**Age** (years)	63.7 ± 12.2	62.9 ± 13.9	66.2 ± 6.9	53.7 ± 8.1	66.6 ± 12.3	49.4 ± 15.5	47.9 ± 13.1	47.1 ± 12.0	38.3 ± 11.4	48.9 ± 14.4
**Female** ─ n(%)	13 (44.8)	8 (72.7)	7 (13.2)	19 (100)	3 (18.8)	28 (63.6)	9 (69.2)	14 (93.3)	5 (50)	35 (67.3)
**Body Mass Index**	26.7 ± 5.4	25.3 ± 5.1	26.8 ± 4.2	28.4 ± 3.3	25.7 ± 3.6	27.0 ± 5.2	27.7 ± 5.3	26.1 ± 6.8	25.9 ± 6.1	27.2 ± 5.8
**Diabetes** ─ n(%)	4 (13.8)	1 (9.1)	8 (15.1)	1 (5.3)	3 (18.8)	0 (0.0)	0 (0.0)	0 (0.0)	0 (0.0)	0 (0.0)
**Hypertension** ─ n(%)	12 (41.4)	2 (18.2)	17 (32.1)	6 (31.6)	7 (43.8)	1 (2.3)	0 (0.0)	0 (0.0)	0 (0.0)	1 (1.9)
**Kidney failure** ─ n(%)	5 (17.2)	0 (0.0)	5 (9.4)	1 (5.3)	1 (6.3)	0 (0.0)	0 (0.0)	0 (0.0)	0 (0.0)	0 (0.0)

Abbreviations: m, male, f, female, DIEP, deep inferior epigastric perforator, SCM, sternocleidomastoid muscle

^*^Plus–minus values are means ± SD

### Time-intensity curves and perfusion parameters

**Fig. 2:**
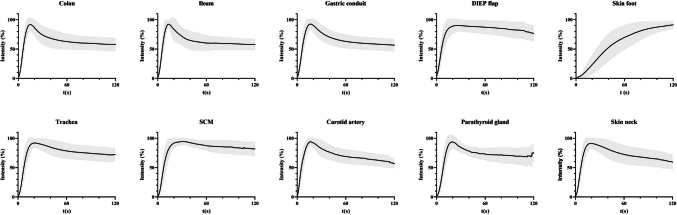
Mean normalized time-intensity curves with standard deviation of the colon, ileum, gastric conduit, DIEP flap, skin foot, trachea, SCM, carotid artery, parathyroid gland and skin neck. Abbreviations: t, time, DIEP, deep inferior epigastric perforator, SCM, sternocleidomastoid muscle

**Fig. 3:**
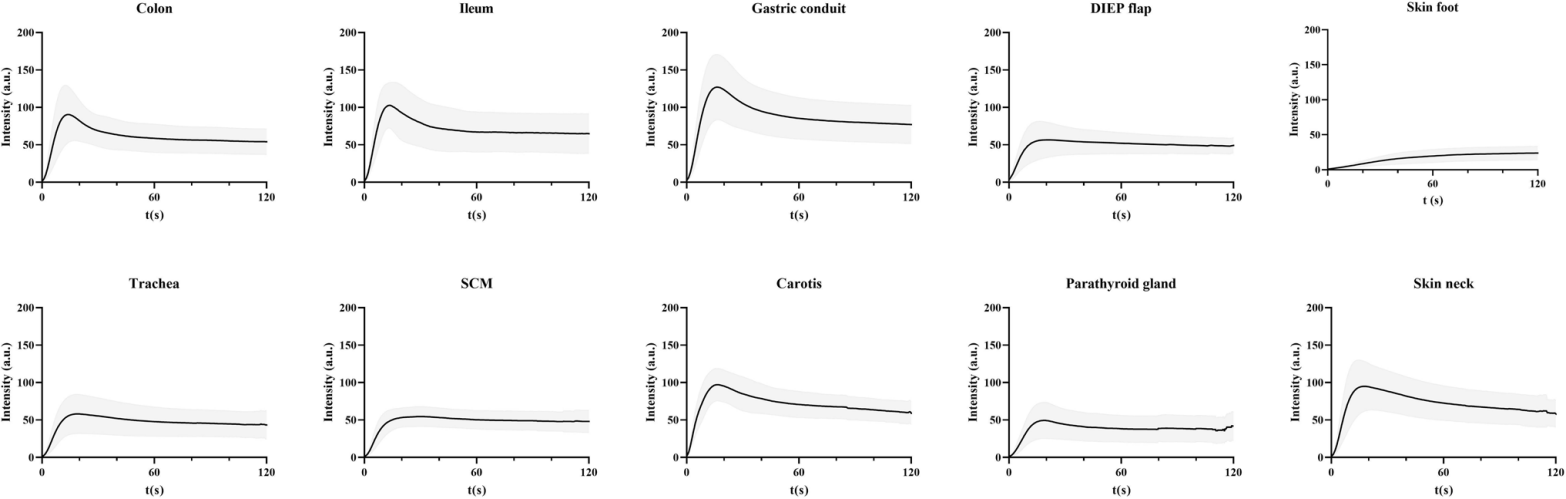
Mean absolute time-intensity curves with standard deviation of the colon, ileum, gastric conduit, DIEP flap, skin foot, trachea, SCM, carotid artery, parathyroid gland and skin neck. Abbreviations: t, time, a.u., arbitrary units, DIEP, deep inferior epigastric perforator, SCM, sternocleidomastoid muscle

**Fig. 4:**
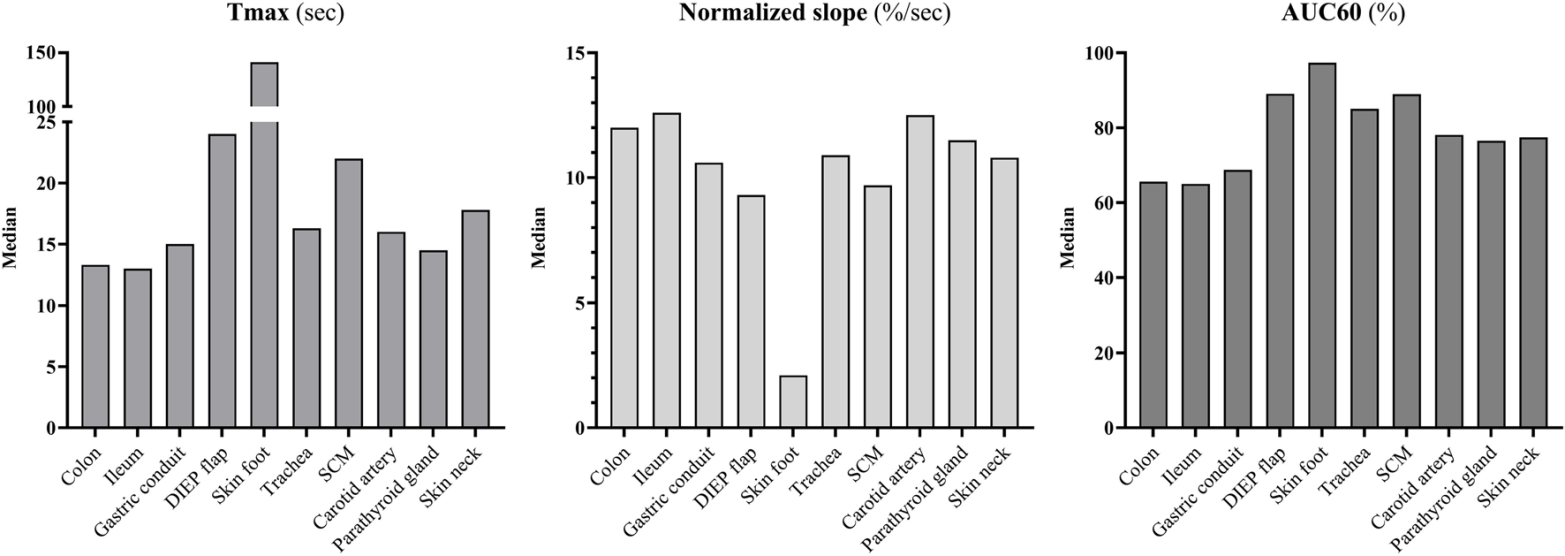
The median values of the quantified perfusion parameters for each tissue type displayed in a bar chart. Abbreviations: sec, seconds, AUC, area under the curve, DIEP, deep inferior epigastric perforator, SCM, sternocleidomastoid muscle

The mean normalized time-intensity curves with standard deviation for all tissues are illustrated in Fig. 2, and the mean absolute time-intensity curves are illustrated in Fig. 3. Parameter extraction of the target tissues is illustrated in Fig. 4 and Table 3. Three distinct perfusion patterns were identified across the analysed tissues, characterized by different inflow and outflow dynamics. Table 4 displays the parameters with overall and pairwise statistical comparison of perfusion metrics across three identified patterns.

**Table 3:** Quantified ICG NIR fluorescence perfusion parameters for each tissue type

	**Colon** (n = 30)	**Ileum** (n = 11)	**Gastric conduit** (n = 53)	**DIEP flap** (n = 29)	**Skin foot** (n = 32)	**Trachea** (n = 44)	**SCM** (n = 13)	**Carotid artery** (n = 15)	**Parathyroid gland** (n = 24)	**Skin neck** (n = 52)
**Tmax** (s) Median (IQR)	13.3 (6.0)	13.0 (6.0)	15.0 (5.5)	24.0 (42.0)	141.4 (112.7)	16.3 (16.3)	22.0 (18.0)	16.0 (4.5)	14.5 (5.1)	17.8 (16.0)
**Slope norm** (%/s) Median (IQR)	12.0 (4.3)	12.6 (5.6)	10.6 (4.1)	9.3 (4.0)	2.1 (0.5)	10.9 (5.7)	9.7 (6.8)	12.5 (5.3)	11.5 (4.4)	10.8 (4.5)
**AUC60** (%) Median (IQR)	65.6 (19.5)	65.0 (11.0)	68.8 (12.4)	89.1 (9.1)	N/A	85.1 (13.8)	89.0 (10.5)	78.1 (14.9)	76.5 (10.9)	77.4 (15.2)

Abbreviations: Tmax, time to max intensity, s, seconds, norm, normalized, AUC, area under the curve, DIEP, deep inferior epigastric perforator, SCM, sternocleidomastoid muscle, IQR, interquartile range, N/A, not applicable

**Table 4:** Statistical comparison of perfusion metrics across three identified perfusion patterns

**Variable**	**Pattern 1**	**Pattern 2**	**Pattern 3**	**Overall comparison**	**Pairwise comparisons** (p-value)
**Tmax (s)** Median (IQR)	15.0 (8.7)	24.0 (37.5)	141.4 (112.7)	p < 0.001	1 vs 2: < 0.001 1 vs 3: < 0.001 2 vs 3: < 0.001
**Slope norm (%/s)** Median (IQR)	11.3 (4.8)	9.5 (4.5)	2.1 (1.4)	p < 0.001	1 vs 2: 0.044 1 vs 3: < 0.001 2 vs 3: < 0.001
**AUC60 (%)** Median (IQR)	74.5 (17.8)	89.0 (9.2)	97.4 (3.0)	p < 0.001	1 vs 2: < 0.001 1 vs 3: < 0.001 2 vs 3: < 0.001

Abbreviations: Tmax, time to max intensity, s, seconds, norm, normalized, AUC, area under the curve, DIEP, deep inferior epigastric perforator, SCM, sternocleidomastoid muscle, IQR, interquartile range

Pattern one was observed in seven out of ten tissues and generally describes rapid inflow, followed by a brief outflow phase that transitioned into a stable state. The rapid inflow was marked by a short time to maximum intensity (Tmax, medians between 13.0 – 17.8 s, total median 15.0 s) and a steep maximum normalised slope ( medians between 12.6 – 0.6%/sec, total median 11.3%/sec). The outflow phase displayed median AUC60 values between 65.0 – 85.1%, with a total median of 74.5%. The tissues demonstrating this pattern included the colon, ileum, gastric conduit, trachea, carotid artery, parathyroid glands, and skin of the neck. Furthermore, the onset of fluorescence intensity in the carotid artery was always faster than any other adjacent tissue, demonstrating a median of 5.0 s (quartiles: 3.0 /5.5).

Pattern two displays a reference curve with a slightly longer inflow period compared to pattern one, followed by a reduced outflow phase. This pattern was observed in the DIEP flap and sternocleidomastoid muscle (SCM) groups. The prolonged inflow phase was marked by longer median Tmax values of 22.0 (SCM) and 24.0 s (DIEP flap), and maximum normalised slopes of 9.3 (DIEP flap) and 9.7%/second (SCM). The outflow phase demonstrated median AUC60 values of 89.0% (SCM) and 89.1% (DIEP flap).

Pattern three demonstrates a distinct reference curve, characterised by a slow and long inflow phase, without reaching a peak and therefore not showing outflow within the first two minutes. This pattern was only observed in the skin of the foot group, and showed a median maximum normalised slope of 2.1%/second and inadequate Tmax and AUC60 values due to not reaching the outflow phase.

The Kruskal–Wallis test revealed significant differences in Tmax, slope norm, and AUC60 across the three perfusion patterns (p < 0.001). Pairwise comparisons using the Mann–Whitney U-test showed the following significant differences: Tmax and AUC60 were significantly different between pattern 1 and pattern 2 (p < 0.001), pattern 1 and pattern 3 (p < 0.001), and pattern 2 and pattern 3 (p < 0.001). Slope norm showed significant differences between pattern 1 and pattern 2 (p = 0.044), as well as between pattern 1 and pattern 3 (p < 0.001), and between pattern 2 and pattern 3 (p < 0.001).

## Discussion

The establishment of reference curves to assess perfusion status using quantified ICG NIR fluorescence imaging is of paramount importance for the implementation of this technique in daily clinical practice. This study provides a first step towards this goal by displaying reference perfusion curves of ten unique, vital target tissues. First, seven out of ten target tissues demonstrated a similar pattern of reference curves. This curve was characterized by three stages, demonstrating a rapid inflow (1), a short period of outflow (2) followed by a gradual or absent outflow (steady state) (3). Outliers of these reference curves were the foot of the skin (gradual, slow inflow), the DIEP flap and the sternocleidomastoid muscle (reduced outflow). Secondly, normalized analysis of the data proved to be the most consistent method for establishing these reference curves, with the normalized slope demonstrating the least variance. Displaying these reference curves for vital tissue perfusion is important since most studies on quantitative ICG NIR fluorescence have predominantly focused on perfusion assessment of tissue at risk [5, 36–38].

In earlier studies on quantified ICG NIR fluorescence imaging, considerable clinical value in multiple perioperative settings was shown, such as intraoperative identification of malperfusion to prevent dreaded ischemia-related complications [19, 23, 37, 39]. Furthermore, pre- and postoperative applications also arise, including the objective evaluation of wound perfusion or quality control for a perfusion-enhancing intervention such as a peripheral bypass [25, 40, 41]. These studies investigated absolute, relative, and time-related parameters, but showed comparable curves of malperfusion, generally described as prolonged inflow and reduced outflow. In the present study, variations in reference curves were observed within tissues, for which several explanations can be given. The only tissue showing an apparent variation of inflow was the skin of the foot, characterised by a slow inflow with low normalized slope, and high Tmax values. This variation could be explained by the distal location of this tissue from the central blood supply. Furthermore, the DIEP flap and SCM showed a noticeable minimal amount of outflow, with a slight delay of inflow. In the DIEP flaps, this finding could be explained by the absence of collateral flow resulting in less outflow of ICG in the flap. For the SCM, the limited outflow could be explained by the retraction of the muscle during surgery and imaging, impeding outflow. The other seven tissues demonstrated comparable reference curves of vital tissue perfusion with rapid inflow, a period of outflow followed by a gradual or absent outflow (steady state), a pattern which has also been described in literature as adequate perfusion [29, 30, 42]. The absolute time-intensity curves displayed in this study showed the anticipated large variation between curves, emphasizing the inaccuracy of intensity-related (absolute) parameters once again [35, 43, 44]. A limitation of this study is the inclusion of data from five separate cohorts with varying imaging protocols (e.g. camera distance, ICG dosage). While normalization reduced variability and allowed for consistent reference curves within this study, it remains uncertain to what extent such variations, or the use of different commercially available devices, may impact fluorescence parameters.

Furthermore, the influence of patient-specific factors like hemodynamics (e.g. blood pressure, cardiac output) on a time-intensity curve is known, however, the limited evidence makes correction for these factors not yet achievable [33, 45–48]. Another limitation of this study is that the vitality of the analysed tissues in this study was presumed, however, not completely guaranteed as it was clinically assessed and most tissues were measured in an operative setting. Additionally, the selection of ROIs involved variation in size and location across different tissue types, tailored to the specific anatomical and physiological characteristics of each tissue. While this approach ensured relevance and consistency, it may also introduce a degree of variability in the results. Since an objective method for intraoperative tissue perfusion assessment is still lacking, the use of clinical assessment as the predominant mode of evaluation seems justifiable. Added to this, the observed minimal variation among the normalized curves within the tissue groups underscores the likelihood of selectively identifying vital tissue.

The significant differences observed in Tmax, Slope norm, and AUC60 across the three perfusion patterns suggest that these patterns reflect distinct physiological states, which could be of clinical relevance in assessing tissue perfusion. The tested tissue groups varied in sample size and the calculated p-values were not corrected for multiple comparisons due to the descriptive nature of this study and small sample sizes, therefore the results should be interpreted accordingly. The clinical utility of these patterns will require further validation, particularly in defining clinically relevant thresholds for differentiating between good and poor perfusion. Therefore, dedicated clinical studies evaluating perfusion curves of tissue in vital and malperfused states, including clinical outcomes, will be needed to address the predictive value of quantified perfusion parameters for assessing the risk of postoperative perfusion related complications. Moreover, better comprehension of tissue perfusion curves in various conditions is needed to determine the technique’s ability to distinguish arterial and venous pathology.

The use of artificial intelligence (AI) is a promising approach to address challenges associated with perfusion assessment using quantitative ICG NIR fluorescence. Despite the clinical promise, the challenges of interpreting results objectively, standardizing imaging protocols, and accounting for patient-specific factors necessitate innovative solutions. AI models have the potential to provide invaluable insights into the detection of perfusion deficits and underlying pathologies [49]. Such AI-driven analysis, possibly combined with other innovative techniques like hyperspectral imaging, could address the full clinical potential of quantitative perfusion assessment, enhancing its precision and reliability in diverse perioperative settings [50]. Building on the insights provided in this study, this technique holds significant potential to become a key tool in preventing complications related to malperfusion in diverse surgical settings, such as anastomotic leakage or skin necrosis. Its clinical adoption could markedly enhance perioperative decision-making and improve patient outcomes.

## Conclusion

This study is the first to describe reference curves for vital tissue perfusion in ten target tissues using quantified NIR fluorescence imaging with indocyanine green, providing a benchmark for adequate perfusion. These findings mark an important step towards the clinical implementation of this technique and highlight its potential to provide surgeons with an objective and reliable tool for perioperative perfusion assessment.

## Data Availability

No datasets were generated or analysed during the current study.

## References

[1] Matienzo D, Bordoni B (2020) Anatomy, blood flow [Internet]. PubMed. Treasure Island (FL): StatPearls Publishing

[2] Robson MC, Steed DL, Franz MG (2001) Wound healing: biologic features and approaches to maximize healing trajectories. Curr Probl Surg 38(2):72–140 11452260 10.1067/msg.2001.111167

[3] Hunt TK (1988) The physiology of wound healing. Ann Emerg Med 17(12):1265–1273 3057943 10.1016/s0196-0644(88)80351-2

[4] Whitney JD (1990) The influence of tissue oxygen and perfusion on wound healing. AACN Clin Issues Crit Care Nurs 1(3):578–584 2223323 10.4037/15597768-1990-3013

[5] McDermott FD, Heeney A, Kelly ME, Steele RJ, Carlson GL, Winter DC (2015) Systematic review of preoperative, intraoperative and postoperative risk factors for colorectal anastomotic leaks. Br J Surg 102(5):462–479 25703524 10.1002/bjs.9697

[6] Fabbi M, Hagens ERC, van Berge Henegouwen MI, Gisbertz SS. Anastomotic leakage after esophagectomy for esophageal cancer: definitions, diagnostics, and treatment. Dis Esophagus. 2021;34(1). 10.1093/dote/doaa039 PMC7801633 32476017

[7] Futier E, Robin E, Jabaudon M, Guerin R, Petit A, Bazin JE et al (2010) Central venous O₂ saturation and venous-to-arterial CO₂ difference as complementary tools for goal-directed therapy during high-risk surgery. Crit Care 14(5):R193 21034476 10.1186/cc9310 PMC3219300

[8] Kroll SS (2000) Fat necrosis in free transverse rectus abdominis myocutaneous and deep inferior epigastric perforator flaps. Plast Reconstr Surg 106(3):576–583 10987463 10.1097/00006534-200009030-00008

[9] Renna MS, Grzeda MT, Bailey J, Hainsworth A, Ourselin S, Ebner M, et al. Intraoperative bowel perfusion assessment methods and their effects on anastomotic leak rates: meta-analysis. Br J Surg. 2023. 10.1093/bjs/znad154 PMC10416696 37253021

[10] Vignali A, Gianotti L, Braga M, Radaelli G, Malvezzi L, Di Carlo V (2000) Altered microperfusion at the rectal stump is predictive for rectal anastomotic leak. Dis Colon Rectum 43(1):76–82 10813128 10.1007/BF02237248

[11] Scheeren TW, Martin K, Maruschke M, Hakenberg OW (2011) Prognostic value of intraoperative renal tissue oxygenation measurement on early renal transplant function. Transpl Int 24(7):687–696 21521383 10.1111/j.1432-2277.2011.01258.x

[12] Ryu HS, Lim SB, Choi ET, Song I, Lee JL, Kim CW et al (2021) Intraoperative perfusion assessment of the proximal colon by a visual grading system for safe anastomosis after resection in left-sided colorectal cancer patients. Sci Rep 11(1):2746 33531598 10.1038/s41598-021-82486-9 PMC7854740

[13] Smit JM, Zeebregts CJ, Acosta R, Werker PMN (2010) Advancements in free flap monitoring in the last decade: a critical review. Plast Reconstr Surg 125(1):177–185 20048610 10.1097/PRS.0b013e3181c49580

[14] Van Den Hoven P, Goncalves LN, Quax PHA, Van Rijswijk CSP, Van Schaik J, Schepers A, et al. Perfusion Patterns in Patients with Chronic Limb-Threatening Ischemia versus Control Patients Using Near-Infrared Fluorescence Imaging with Indocyanine Green. Biomedicines. 2021;9(10). 10.3390/biomedicines9101417 PMC8533354 34680534

[15] St John A, Caturegli I, Kubicki NS, Kavic SM. The Rise of Minimally Invasive Surgery: 16 Year Analysis of the Progressive Replacement of Open Surgery with Laparoscopy. Jsls. 2020;24(4). 10.4293/JSLS.2020.00076 PMC7810432 33510568

[16] Karliczek A, Harlaar NJ, Zeebregts CJ, Wiggers T, Baas PC, van Dam GM (2009) Surgeons lack predictive accuracy for anastomotic leakage in gastrointestinal surgery. Int J Colorectal Dis 24(5):569–576 19221768 10.1007/s00384-009-0658-6

[17] Vidal Fortuny J, Sadowski SM, Belfontali V, Guigard S, Poncet A, Ris F et al (2018) Randomized clinical trial of intraoperative parathyroid gland angiography with indocyanine green fluorescence predicting parathyroid function after thyroid surgery. Br J Surg 105(4):350–357 29405252 10.1002/bjs.10783 PMC6084300

[18] Desai ND, Miwa S, Kodama D, Cohen G, Christakis GT, Goldman BS et al (2005) Improving the Quality of Coronary Bypass Surgery With Intraoperative Angiography: Validation of a New Technique. J Am Coll Cardiol 46(8):1521–1525 16226178 10.1016/j.jacc.2005.05.081

[19] Driessen C, Arnardottir TH, Lorenzo AR, Mani MR (2020) How should indocyanine green dye angiography be assessed to best predict mastectomy skin flap necrosis? A systematic review. J Plast Reconstr Aesthet Surg 73(6):1031–1042 32245733 10.1016/j.bjps.2020.02.025

[20] Gerken ALH, Nowak K, Meyer A, Weiss C, Krüger B, Nawroth N et al (2022) Quantitative Assessment of Intraoperative Laser Fluorescence Angiography With Indocyanine Green Predicts Early Graft Function After Kidney Transplantation. Ann Surg 276(2):391–397 33394595 10.1097/SLA.0000000000004529 PMC9259036

[21] Ladak F, Dang JT, Switzer N, Mocanu V, Tian C, Birch D et al (2019) Indocyanine green for the prevention of anastomotic leaks following esophagectomy: a meta-analysis. Surg Endosc 33(2):384–394 30386983 10.1007/s00464-018-6503-7

[22] Noltes ME, Metman MJH, Heeman W, Rotstein L, van Ginhoven TM, Vriens MR et al (2021) A Novel and Generic Workflow of Indocyanine Green Perfusion Assessment Integrating Standardization and Quantification Toward Clinical Implementation. Ann Surg 274(6):e659–e663 34145192 10.1097/SLA.0000000000004978

[23] Pollmann L, Juratli M, Roushansarai N, Pascher A, Hölzen JP. Quantification of Indocyanine Green Fluorescence Imaging in General, Visceral and Transplant Surgery. J Clin Med. 2023;12(10). 10.3390/jcm12103550 PMC10219543 37240657

[24] Van Den Hoven P, Osterkamp J, Nerup N, Svendsen MBS, Vahrmeijer A, Van Der Vorst JR et al (2023) Quantitative perfusion assessment using indocyanine green during surgery - current applications and recommendations for future use. Langenbecks Arch Surg 408(1):67 36700999 10.1007/s00423-023-02780-0 PMC9879827

[25] Wermelink B, Ma KF, Haalboom M, El Moumni M, de Vries JPM, Geelkerken RH (2021) A Systematic Review and Critical Appraisal of Peri-Procedural Tissue Perfusion Techniques and their Clinical Value in Patients with Peripheral Arterial Disease. Eur J Vasc Endovasc Surg 62(6):896–908 34674935 10.1016/j.ejvs.2021.08.017

[26] Armstrong G, Croft J, Corrigan N, Brown JM, Goh V, Quirke P et al (2018) IntAct: intra-operative fluorescence angiography to prevent anastomotic leak in rectal cancer surgery: a randomized controlled trial. Colorectal Dis 20(8):O226–O234 29751360 10.1111/codi.14257 PMC6099475

[27] Meijer RPJ, Faber RA, Bijlstra OD, Braak J, Meershoek-Klein Kranenbarg E, Putter H et al (2022) AVOID; a phase III, randomised controlled trial using indocyanine green for the prevention of anastomotic leakage in colorectal surgery. BMJ Open 12(4):e051144 35365509 10.1136/bmjopen-2021-051144 PMC8977759

[28] Watanabe J, Takemasa I, Kotake M, Noura S, Kimura K, Suwa H et al (2023) Blood Perfusion Assessment by Indocyanine Green Fluorescence Imaging for Minimally Invasive Rectal Cancer Surgery (EssentiAL trial): A Randomized Clinical Trial. Ann Surg 278(4):e688–e694 37218517 10.1097/SLA.0000000000005907 PMC10481925

[29] Faber RA, Tange FP, Galema HA, Zwaan TC, Holman FA, Peeters K, et al. Quantification of indocyanine green near-infrared fluorescence bowel perfusion assessment in colorectal surgery. Surg Endosc. 2023. 10.1007/s00464-023-10140-8 PMC10462565 37286750

[30] Galema HA, Faber RA, Tange FP, Hilling DE, van der Vorst JR (2023) A quantitative assessment of perfusion of the gastric conduit after oesophagectomy using near-infrared fluorescence with indocyanine green. Eur J Surg Oncol 49(5):990–995 36914531 10.1016/j.ejso.2023.02.017

[31] Nerup N, Svendsen MBS, Svendsen LB, Achiam MP (2020) Feasibility and usability of real-time intraoperative quantitative fluorescent-guided perfusion assessment during resection of gastroesophageal junction cancer. Langenbecks Arch Surg 405(2):215–222 32281020 10.1007/s00423-020-01876-1

[32] Goncalves LN, van den Hoven P, van Schaik J, Leeuwenburgh L, Hendricks CHF, Verduijn PS, et al. Perfusion Parameters in Near-Infrared Fluorescence Imaging with Indocyanine Green: A Systematic Review of the Literature. Life (Basel). 2021;11(5). 10.3390/life11050433 PMC8151115 34064948

[33] Vaassen HGM, Wermelink B, Geelkerken RH, Lips DJ (2022) Fluorescence-Based Quantification of Gastrointestinal Perfusion: A Step Towards an Automated Approach. J Laparoendosc Adv Surg Tech A 32(3):293–298 33739876 10.1089/lap.2021.0102

[34] Tange FP, Verduijn PS, Sibinga Mulder BG, van Capelle L, Koning S, Driessen C et al (2023) Near-infrared fluorescence angiography with indocyanine green for perfusion assessment of DIEP and msTRAM flaps: A Dutch multicenter randomized controlled trial. Contemp Clin Trials Commun 33:101128 37091505 10.1016/j.conctc.2023.101128 PMC10119502

[35] Van Den Hoven P, Tange F, Van Der Valk J, Nerup N, Putter H, Van Rijswijk C et al (2023) Normalization of Time-Intensity Curves for Quantification of Foot Perfusion Using Near-Infrared Fluorescence Imaging With Indocyanine Green. J Endovasc Ther 30(3):364–371 35236169 10.1177/15266028221081085 PMC10209496

[36] Ma KF, Kleiss SF, Schuurmann RCL, Bokkers RPH, Ünlü Ç, De Vries JPM (2019) A systematic review of diagnostic techniques to determine tissue perfusion in patients with peripheral arterial disease. Expert Rev Med Devices 16(8):697–710 31340684 10.1080/17434440.2019.1644166

[37] van den Hoven P, Ooms S, van Manen L, van der Bogt KEA, van Schaik J, Hamming JF et al (2019) A systematic review of the use of near-infrared fluorescence imaging in patients with peripheral artery disease. J Vasc Surg 70(1):286–97.e1 31230648 10.1016/j.jvs.2018.11.023

[38] Lauritzen E, Damsgaard TE (2021) Use of Indocyanine Green Angiography decreases the risk of complications in autologous- and implant-based breast reconstruction: A systematic review and meta-analysis. J Plast Reconstr Aesthet Surg 74(8):1703–1717 33931326 10.1016/j.bjps.2021.03.034

[39] Van Den Hoven P, Van Den Berg SD, Van Der Valk JP, Van Der Krogt H, Van Doorn LP, Van De Bogt KEA et al (2022) Assessment of Tissue Viability Following Amputation Surgery Using Near-Infrared Fluorescence Imaging With Indocyanine Green. Ann Vasc Surg 78:281–287 34182113 10.1016/j.avsg.2021.04.030

[40] Tange FP, van den Hoven P, van Schaik J, Schepers A, van der Bogt KEA, van Rijswijk CSP, et al. Near-Infrared Fluorescence Imaging With Indocyanine Green to Predict Clinical Outcome After Revascularization in Lower Extremity Arterial Disease. Angiology. 2023:33197231186096. 10.1177/00033197231186096 PMC11375904 37358400

[41] Van den Hoven P, F SW, Van De Bent M, Goncalves LN, Ruig M, S DVDB, et al. Near-infrared fluorescence imaging with indocyanine green for quantification of changes in tissue perfusion following revascularization. Vascular. 2022;30(5):867–73. 10.1177/17085381211032826 34320878

[42] Joosten JJ, Slooter MD, van den Elzen RM, Bloemen PR, Laméris W, de Bruin DM et al (2023) Understanding fluorescence time curves during ileal pouch-anal anastomosis with or without vascular ligation. Surg Endosc 37(7):5086–5093 36917344 10.1007/s00464-023-09921-y PMC10322949

[43] Lütken CD, Achiam MP, Svendsen MB, Boni L, Nerup N (2020) Optimizing quantitative fluorescence angiography for visceral perfusion assessment. Surg Endosc 34(12):5223–5233 32696147 10.1007/s00464-020-07821-z

[44] Slooter MD, Mansvelders MSE, Bloemen PR, Gisbertz SS, Bemelman WA, Tanis PJ, et al. Defining indocyanine green fluorescence to assess anastomotic perfusion during gastrointestinal surgery: systematic review. BJS Open. 2021;5(2). 10.1093/bjsopen/zraa074 PMC8271268 33893811

[45] Son GM, Nazir AM, Yun MS, Lee IY, Im SB, Kwak JY, et al. The Safe Values of Quantitative Perfusion Parameters of ICG Angiography Based on Tissue Oxygenation of Hyperspectral Imaging for Laparoscopic Colorectal Surgery: A Prospective Observational Study. Biomedicines. 2023;11(7). 10.3390/biomedicines11072029 PMC10377371 37509667

[46] Son GM, Kim TU, Park BS, Jung HJ, Lee SS, Yoon JU et al (2019) Colonic hypoperfusion following ligation of the inferior mesenteric artery in rectosigmoid colon cancer patients. Ann Surg Treat Res 97(2):74–82 31388509 10.4174/astr.2019.97.2.74 PMC6669131

[47] Elliott JT, Addante RR, Slobegean GP, Jiang S, Henderson ER, Pogue BW et al (2020) Intraoperative fluorescence perfusion assessment should be corrected by a measured subject-specific arterial input function. J Biomed Opt 25(6):1–14 32519522 10.1117/1.JBO.25.6.066002 PMC7282620

[48] Osterkamp J, Strandby R, Nerup N, Svendsen M, Svendsen L, Achiam M (2021) Quantitative fluorescence angiography detects dynamic changes in gastric perfusion. Surg Endosc 35(12):6786–6795 33258036 10.1007/s00464-020-08183-2

[49] Cahill RA, O’Shea DF, Khan MF, Khokhar HA, Epperlein JP, Mac Aonghusa PG et al (2021) Artificial intelligence indocyanine green (ICG) perfusion for colorectal cancer intra-operative tissue classification. Br J Surg 108(1):5–9 33640921 10.1093/bjs/znaa004

[50] Pfahl A, Radmacher GK, Kohler H, Maktabi M, Neumuth T, Melzer A et al (2022) Combined indocyanine green and quantitative perfusion assessment with hyperspectral imaging during colorectal resections. Biomed Opt Express 13(5):3145–3160 35774324 10.1364/BOE.452076 PMC9203086

